# Early identification of permanent maxillary canine impaction: A radiographic comparative study in a Mexican population

**DOI:** 10.4317/jced.55285

**Published:** 2019-03-01

**Authors:** Karen Alejos-Montante, Alán Martínez-Zumarán, Gabriela Torre-Delgadillo, Miguel-Ángel Rosales-Berber, Arturo Garrocho-Rangel, Amaury Pozos-Guillén

**Affiliations:** 1 DDS, Pediatric Dentistry Postgraduate Program, Faculty of Dentistry, San Luis Potosi University, San Luis Potosí, SLP, México; 2 DDS, MSc, Orthodontics Postgraduate Program, Faculty of Dentistry, San Luis Potosi University, San Luis Potosí, SLP, México; 3 DDS, MSc, Pediatric Dentistry Postgraduate Program, Faculty of Dentistry, San Luis Potosi University, San Luis Potosí, SLP, México; 4 DDS, MSc, Pediatric Dentistry Postgraduate Program, Faculty of Dentistry, San Luis Potosi University, San Luis Potosí, SLP, México; 5 DDS, MSc, PhD, Pediatric Dentistry Postgraduate Program, Faculty of Dentistry, San Luis Potosi University, San Luis Potosí, SLP, México; 6 DDS, MSc, PhD, Pediatric Dentistry Postgraduate Program, Faculty of Dentistry, San Luis Potosi University, San Luis Potosí, SLP, México

## Abstract

**Background:**

Opportune diagnosis, prediction, and interceptive treatment of permanent maxillary canine (PMC) impaction is fundamental for pediatric dentists and orthodontists. In children and young adolescents, diagnostic information obtained from a panoramic radiograph is valuable for the overview and prediction of a potential PMC ectopic eruption into the oral cavity. The aim of the present study was to calculate and compare the prevalence of impaction of PMC in a Mexican pediatric sample (7 to 13 years old), through the use of the Ericson & Kurol (EK/L) and the Power & Short (PS) measurement analyses performed on panoramic radiographs.

**Material and Methods:**

This investigation was a cross-sectional study performed on 515 panoramic radiographs, which were evaluated to assess the intraosseous position of right and left PMC, from patients who had attended our clinic between 2010 and 2017. Both analytical methods were applied on the same radiography. Outcomes from both analysis methods were expressed dichotomously (impacted or non-impacted). Thus, prevalence was calculated from each method, and the difference between them was verified through the Pearson’s Chi-square test.

**Results:**

No statistical difference could be detected between both prevalence rates (*p* = 0.475). It was found a significant predilection of the condition to the female sex (*p* = 0.034). Further, the two radiographic methods employed here were highly concordant each other (kappa = 0.92).

**Conclusions:**

Through the EK/L method a PMC prevalence of 5.64% (95% CI = 3.66, 7.62) was obtained, while the PS Method the prevalence was 8.83% (95% CI = 6.38, 11.28). In addition, a significant predilection of canine impaction to the female gender was found.

** Key words:**Maxillary canine impaction, prevalence, radiographic analyses.

## Introduction

Impacted teeth are defined as those with delayed eruption or that are not expected to erupt in a correct position based on clinical or radiographic assessment. After the third molar, the permanent maxillary canine (PMC) is the most frequently impacted tooth, with a reported prevalence reported of 1 to 3%, more frequently in the female (1-4). This condition is due to an extended development period of the tooth and to individual variations in the tortuous path of eruption patterns and timing, caused by hard or soft tissue obstruction, before reaching the full occlusion in the oral cavity (5,6). Therefore, ectopic eruption of PMC may be inadvertently overlooked during the mixed dentition (7).

Patients with canine impactions may experience longer treatment times, depending on the position of the tooth in relation to the occlusal plane (buccal or palatal) (3). Different studies have reported that 85% of impacted PMC are located palatally, with ratios of 3:1 up to 12:1 regarding the buccal position (8-11). Additionally, it has been suggested that females exhibit until twice palatally impacted canines than males (12). Impaction of PMC is a clinical problem which may cause detrimental effects such as ectopic eruption of the tooth, resorption of incisor roots, or canine ankyloses (3). Therefore, it is paramount to the pediatric dentistry practitioner the opportune identification and interceptive management of the potential impaction of unerupted PMC, because orthodontic/orthopedic treatment may be affected or delayed (7,13). Moreover, treating a malocclusion with one or more impacted canines takes longer than treating a similar malocclusion without an impaction (14).

Thus, it is recommended the regular inspection and palpation of the canine region from the age of 8 years (6). Similarly, early diagnosis of PMC impaction or abnormal displacement regarding the surrounding structures can be performed by means of specific measurement analyses on the panoramic radiography during the mixed dentition stage (15,16). These methods take into consideration two prediction factors: the mesiodistal location of the canine crown and the angulation of the tooth (3). Two of the most popular analyses employed for these purposes are the Ericson and Kurol (modified by Lindauer et al.) and the Power and Short methods (17-19). The first method concluded that palatal impaction of PMC could be prevented by the opportune extraction of the corresponding deciduous canine (10,16); in the second one, authors found that when the PMC is angled more than 31° to the midline, this extraction procedure is justified (3,19).

The first aim of the current cross-sectional study was to determine the prevalence of impaction of PMC in a representative pediatric sample of Mexican origin, by using both the Ericson and Kurol [modified by Lindauer *et al.*] and the Power and Short measurement analyses on the same panoramic radiograph. The second aim was to test the null hypothesis that the numerical information provided to estimate the potential impaction of PMC is not significantly different when these two methods are compared.

## Material and Methods

The present comparative cross-sectional study was carried out in the Pediatric Dentistry Postgraduate Program Clinic (Faculty of Dentistry, San Luis Potosi University, México) and approved by the Ethical Research Committee (Code CEIFE-031-017). A total of 815 available good-quality panoramic radiographs were evaluated to assess the intraosseous position of right and left PMC, from nonsyndromic patients in mixed dentition stage (7 to 13 years old and with permanent upper incisors fully erupted and unerupted PMC), who had attended the clinic during the years between 2010 and 2017; they had no antecedents of orthodontic/orthopedic treatment. Exclusion criteria were presence of severe maxillary anterior crowding, absence of one or two permanent lateral incisors, and PMC with less than one third of root development. Regarding the sample size calculation, we considered an expected prevalence of PMC between 3 to 5%; according to Naing et al’s method, this prevalence corresponds to a precision of 0.015, and thus, to a resultant n of 493 participants, as a minimum (20).

All radiographs were taken on the same radiograph machine examined in a darkened room, employing a negatoscope. Radiographs were traced superimposing a matted acetate paper with a 0.5 mm fine lead pencil. A single trained evaluator (K.D.A.M.) was precalibrated in fifteen randomly panoramic radiographs for intra- and inter-observer agreement through the Cohen’s kappa test (0.88 and 0.91, respectively); for each calibration procedure the same radiograph set was evaluated twice (sixty evaluations in total). Measurements, chronological age and sex were collected and entered into an electronic spreadsheet.

For these purposes, the Ericson and Kurol [modified by Lindauer *et al.*] (EK/L) and the Power and Short (PS) geometric measurement analyses were employed; both methods were applied in every radiograph. Following, these methods are briefly described.

**Figure 1:**
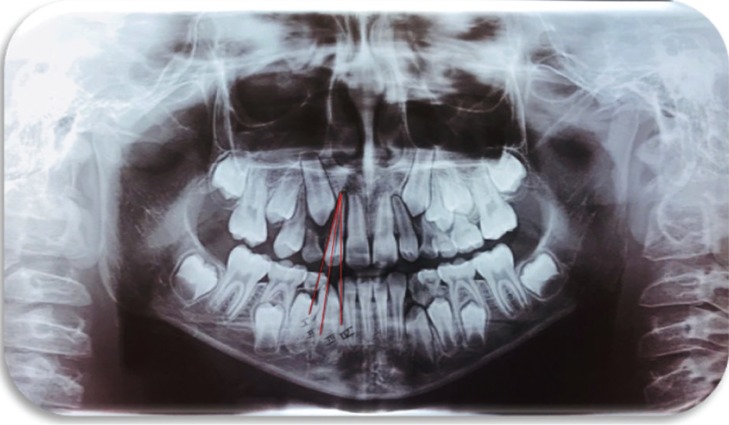
Ericson & Kurol method.

EK/L method is also known as “sectorial method” (17,18). Three lines (distal, central, and mesial) were drawn, tangent to the root and crown contour of the neighbor permanent lateral incisor (Fig. 1). Four vertical sectors were created: sector I was distal to the distal line, corresponding to the primary canine; sector II was the area between distal and central lines; sector III includes the area from the central line to the mesial line; and, sector IV is the space mesial to sector III. The mesiodistal position of the unerupted PMC was classified according to the sector in which the canine cusp tip was located (PMC-I, PMC-II, PMC-III, PMC-IV); PMC-I and PMC-II were rated as “low risk of impaction” (LRI), and PMC-III and PMC-IV as “high risk of impaction” (HRI).

**Figure 2:**
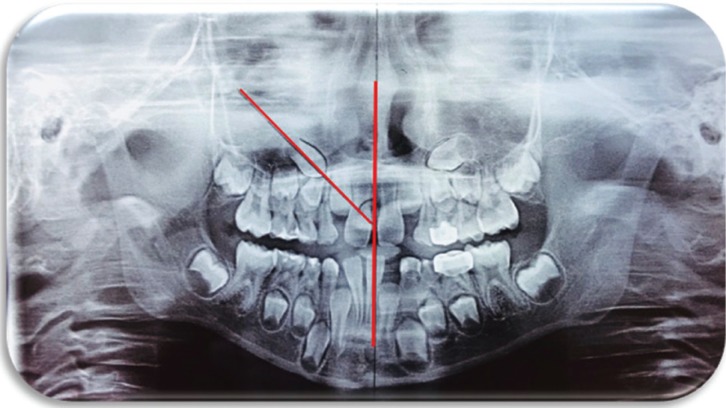
Power & Short method.

PS method, the midline was first drawn passing through the intermaxillary suture, anterior nasal spine, nasal septum, and internasal suture (3,16,19). Then, a perpendicular line was drawn on it, which served as the horizontal reference plane. A mesial angular measurement was obtained from this midline to the longitudinal axis of the unerupted PMC (Fig. 2). Angles between 0-30° were rated as LRI and those more than 31° as HRI.

A brief statistical description of the pediatric sample was performed. Outcomes from both analysis methods were expressed dichotomously, namely percentages of LRI or HRI. Each percentage of HRI was considered as the equivalent of the prevalence of canine impaction. Thus, prevalence (and its 95% Confidence Interval) was calculated from each method, and the difference between prevalences was statistically verified through the Pearson’s Chi-square test, with a p value set at 0.05. Finally, both radiographic methods were compared for statistical concordance –beyond the chance– through the Cohen’s kappa test. All statistical procedures were carried out through the SPSS v. 15 software.

## Results

In total, 815 panoramic radiographs were initially evaluated, but only 515 met the prespecified inclusion criteria. Thus, 1030 PMC were analyzed by means of the two measurement methods. The sample of radiographs was obtained from patients aged between 7 to 13 years, 239 (46.4%) were females and 276 males (56.6%). In both methods, the right side exhibited a high prevalence of tooth impactions and the condition was bilateral in 13.04% of cases.

Forty six PMC were classified as HRI through the EK/L method (25 in females and 21 in males), with a prevalence of 5.64% (95% CI = 3.66, 7.62). While the PS method was used, 72 (40 in females and 32 in males) HRI were found, with a prevalence 8.83% (95% CI = 6.38, 11.28). When both prevalences were compared, no statistical difference could be detected between them (*p* = 0.475). Compared by gender, female patients exhibited a higher prevalence of PMC impaction (*p* = 0.034) ( Table 1). The statistical concordance between the two radiographic methods was high (kappa = 0.92).

**Table 1:**
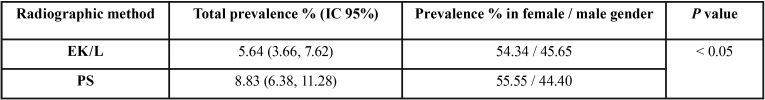
Total prevalences and prevalence for gender of PMC in the studied sample.

## Discussion

The present study was undertaken to evaluate the prevalence of impaction of PMC in a Mexican sample of children aged between 7 to 13 years, according to two well-recognized radiographic measurement analyses. The PMC impaction prevalence rates reported here (between more than 5.5 and almost 9 percent) are as high as those reported from previous studies, also in a Mexican sample (21), and others carried out in Hungary (22), China (23), Greek (24), and India (25). However, when comparing our data with other three studies performed on similar Latin-American populations, these rates are significantly higher (5,26,27), with reported prevalence rates of 3.4, 2.9, and 2.3, respectively.

Palatal impaction has been reported much more prevalent than labial impaction, and unilateral impaction is more common than bilateral (10,16). Principal etiological factors involved in canine impaction include arch space deficiency, trauma, abnormal and prolonged retention of primary canine, premature root closure, cysts, odontomas, rotation of tooth bud, and eruption disturbances. About this, in 1976, Moyers and colleagues described clearly the long and complex eruption route of the PMC in this manner: “At the age of 3 it is high in the maxilla, with its crown directed mesially and somewhat lingually. It moves towards the occlusal plane, gradually uprighting itself until it seems to strike the distal aspect of root of the lateral incisor. It then seems to be deflected to a more vertical position; however, it often erupts into the oral cavity with a marked mesial inclination” (11).

As previously mentioned, the precise diagnosis of a PMC is based on a combination of careful clinical and exhaustive radiographic assessments in the panoramic image (3,6,10). Clinically, a normal maxillary canine can be palpated with index fingers high in the buccal sulcus, above the primary canine root, manifested by the presence of an evident bulge (10). Therefore, this process it is strongly recommended since the age of 7 or 8 years. Regarding to this procedure, diverse authors have stated that the absence of either clinical mobility of the primary canine or the palpation of a labial bulge beyond 10 to 13 years of age are strongly indicative of PMC impaction, which must be confirmed radiographically; conversely, in patients below 10 with a potential for intraosseous malposition may later exhibit a normal eruption path (10,16,28). Therefore, very early radiographic examination –before 7 or 8 years old– is not advisable for predicting the final path of eruption of PMC; a close clinical supervision may be sufficient in this age group (28).

Impaction of maxillary canines is considered as a common clinical anomaly encountered in children, which management requires an interdisciplinary approach (11). Management of this anomaly is associated with prolonged treatment time and increased inherent costs (3,13,14). Interceptive treatment consisting in the primary canine extraction combined with creation of space in the arch, for example, with maxillary expansion, is usually the first choice in order to guide the canine into a normal position. If these options fail, the surgical exposure and orthodontic appliances are indicated to bring the canine into the dental arch (2,4,14).

One possible limitation of the radiographic analyses employed in the present study was that, panoramic radiographs are two-dimensional images, and thus, lack information about the labio-palatal position of the PMC and, in its case, of the root resorption of lateral incisors. Also, it may be problematic to distinguish specific structures based on conventional 2D radiographs, which may lead to some misinterpretations, for example, whether the PMC are truly impacted (14). On the other hand, panoramic radiograph analyses as diagnosis tools for localizing PMC have demonstrated high sensitivity, specificity, and positive/negative predictive values (29,30). Katnelson and colleagues, evaluated the position (buccal or palatal) of 130 PMC, based on the measuring of the mean inclination angle of the tooth to a horizontal reference line; from a receiver-operator characteristic curve, they found that inclination angles greater than 65° were 26.6 times more likely to be positioned in a buccal position (29).

The findings found in the present study, confirm the recommendation of periodic clinical and radiographic assessment, initiating at ages of 7 or 8 years, using any of the radiographic analyses employed here, in order to opportunely diagnose potentially impacted PMC, and thus to avoid future associated complications.

## Conclusions

The EK/L method determined a PMC prevalence on panoramic radiographs of 5.64% (95% CI = 3.66, 7.62), while in the PS Method the prevalence was 8.83% (95% CI = 6.38, 11.28). In addition, a significant predilection of canine impaction to the female gender was found.
